# Overview of Trends in the Application of Metagenomic Techniques in the Analysis of Human Enteric Viral Diversity in Africa’s Environmental Regimes

**DOI:** 10.3390/v10080429

**Published:** 2018-08-14

**Authors:** Cecilia Oluseyi Osunmakinde, Ramganesh Selvarajan, Timothy Sibanda, Bhekie B Mamba, Titus A.M Msagati

**Affiliations:** 1 Nanotechnology and Water Sustainability Research Unit, College of Science, Engineering and Technology, University of South Africa-Science Campus, Florida 1710, South Africa; coadebo@yahoo.com (C.O.O.); mambabb@unisa.ac.za (B.B.M.); 2 Department of Environmental Sciences, College of Agriculture and Environmental Sciences, University of South Africa-Science Campus, Florida 1710, South Africa; ramganesh.presidency@gmail.com (R.S.); timsibanda@gmail.com (T.S.)

**Keywords:** metagenomics, enteric viruses, viral diversity, virus identification

## Abstract

There has been an increase in the quest for metagenomics as an approach for the identification and study of the diversity of human viruses found in aquatic systems, both for their role as waterborne pathogens and as water quality indicators. In the last few years, environmental viral metagenomics has grown significantly and has enabled the identification, diversity and entire genome sequencing of viruses in environmental and clinical samples extensively. Prior to the arrival of metagenomics, traditional molecular procedures such as the polymerase chain reaction (PCR) and sequencing, were mostly used to identify and classify enteric viral species in different environmental milieu. After the advent of metagenomics, more detailed reports have emerged about the important waterborne viruses identified in wastewater treatment plant effluents and surface water. This paper provides a review of methods that have been used for the concentration, detection and identification of viral species from different environmental matrices. The review also takes into consideration where metagenomics has been explored in different African countries, as well as the limitations and challenges facing the approach. Procedures including sample processing, experimental design, sequencing technology, and bioinformatics analysis are discussed. The review concludes by summarising the current thinking and practices in the field and lays bare key issues that those venturing into this field need to consider and address.

## 1. Introduction

By definition, metagenomics refers to the direct study of microbes’ genetic material in their natural habitat [1,2]. It is an approach that allows for the identification of both cultivable and uncultivable microbes in a mixed community, based on a genomic technique [2,3,4]. The application of metagenomics was first reported in the late 19th century, when Norman Pace’s laboratory conceived the notion of gross extraction of deoxyribonucleic acid (DNA) from a sample with a mixture of nucleic acid. Since then, significant progress has been made in metagenomics in different types of environmental compartments. The presence of nucleic acid has been identified from diverse environment such as soil, ocean, sediment, groundwater, as well as in clinical samples. Currently, metagenomics studies are being explored in marine environments [4,5,6] and in disease diagnosis [7,8] to name but a few. In addition, other metagenomics studies have been conducted in terms of human and animal genetics [9,10,11], veterinary medicine [12,13,14] the textile industry [2,15], food and pharmaceutical products [16], biosensors [17], and agriculture biotechnology [18]. Metagenomic approaches have become an emerging and alternative tool for the study of viral taxonomy and varieties in the functional compositions within the aquatic environments, via next generation sequencing (NGS) technology [19]. The merits and opportunities obtained from metagenomics include the study and discovery of microbial genomes that could not be determined previously, due to certain cultivation difficulties.

**Figure 1:**
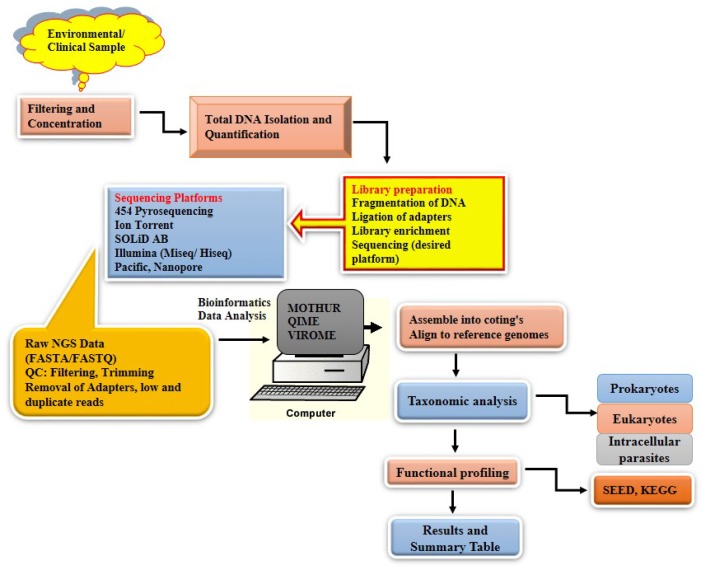
Schematic diagram of the experimental workflow of different next generation sequencing (NGS) platforms. (KEGG—Kyoto Encyclopedia of Genes and Genomes; SEED—Database contains all publicly available genome sequences).

NGS is a genomic sequencing technique that enables massive parallel sequencing of the small fragments of the entire genetic material obtained from a microbial community, which generates massive data output in only one run, through the use of a high-throughput instrumentation [1,20] NGS sequencing technologies are spread out under different sequencing platforms, though they follow the same experimental work flow [21,22]. The general experimental workflow for metagenomics study applying NGS is presented in Figure 1.

Metagenome analysis by NGS involves several distinct steps, with the most important step being the extraction of high quality total DNA from a sample. This is followed by fragmentation and appropriate adapter ligation on the desired platform for the library preparation and sequencing [1,23]. The analysis of the pieces of fragments and voluminous data generated from the different high-throughput platforms, is done by sorting and assembling them into contigs through bioinformatics tools, which is usually the most challenging and tedious task when undertaking metagenomics projects [1,24]. The filtering of the raw sequences is the first step before downstream analysis, and this is achieved through the elimination of low-quality reads and adapters which were attached to the primer sequences. For instance tools like Btrim, Cutadapt, AdapterRemoval, FASTX toolkit and Krakeen are very efficient tools for filtering of low-quality read sequences, removal of adapters and barcodes and for a detailed quality control on raw reads [25]. The genomes are assembled together to form a contigs using various assembly tools. Over the years, quite a lot of assembly tools or algorithms have been developed that depend solely on specific parameters for the assembling of the raw reads [1,22,24]. The assembling of the raw reads are either through a reference-guide genome assembly or through a *de novo* genome assembly [26]. Assembling tools such as SSAKE, Edena, Velvet, VCAKE, SOAPdenovo, De Bruijn graph-based assemblers and the latest addition to the group EULER has been used to assemble reads each with its own strength and weakness [27,28,29]. After the assembling, the sequences are mapped or aligned against a reference database that contains genomes that are specific to taxonomic classification. In this regard, tools and software packages such as Newbler, MIRA, AMOS, Botiwe, BLAT, Bfast, BWA, NovoAlign and MetaAMOS are commonly used in metagenomics for performing referenced-based assemblies [26,30]. The taxonomic designations and phylogenetic tree analysis of the organisms are done using sequences already deposited on the public sequence database that are specifically designed for the nucleotide and protein translations, with examples such as the European molecular biology laboratory (EMBL), GenBank, Basic Local Alignment Search Tool (BLAST), Reference Sequence (RefSeq) and the SWISS-PROT [31,32]. Numerous tool programme and software packages such as ARB [33], Naïve Bayes Classification (NBC) [34,35], k-SLAM [36], CLARK [34,36] MEGAN [34], SILVAngs [26], MetaPhlAn [34], Kraken [34,36,37], CARMA [26], interpolated Markov models [36,38], to name just a few, have been used. Bioinformatics tools are playing significant roles in all fields, in medicines for the treatment and cure of some notable diseases [31,32], drug discovery and testing [39,40], microbial genome [31,32], gene discovery and therapy [31,32,41], agriculture [31,42], antibiotic resistance [43,44,45], alternative energy source [32] and also in the study of climate changes [45].

Viruses undergo a vital part in the environment such as recycling of carbon in the marine environment, infecting and destroying bacteria in aquatic microbial communities [46,47,48]. The existence and great quantity of viruses on Earth has been pointed out, hence this has increased awareness about their wide diversity [46]. Generally, viruses are known to be intracellular parasites made up of a nucleic acid core. The viruses are enclosed by a protein coat known as capsid that is capable of replication through adsorption, penetration, uncoating, viral genome replication, maturation and release, which is only possible within the living cells of bacteria, animals and plants [49,50]. Viruses depend on their host’s cells’ metabolism, for energy, enzymes, and precursors, in order to replicate and multiply. A virion is made up of a protein coat and genomic information, encoded in DNA/RNA. Viruses are categorized on the basis of their dimension, mode of replication, chemical configuration and morphology [50], as well as to establish whether they are single stranded or double stranded, linear or circular [49]. The main function of the virion is to deliver its genome into the host cell for expression and replication of itself [49]. Viruses are host specific and they depend on the host organism to supply the complex metabolic and biosynthetic machinery of eukaryotic or prokaryotic cells [50]. For viruses to propagate successfully in any cell, the virion must be able to identify and bind to its cellular receptor, as well as replicate its own genome.

Studies have shown that the most prominent viral species within the aquatic ecosystem are human enteric viruses (HEV) [51,52,53], which have the ability to survive in the intestinal tract of humans and animals [54,55]. At present over 140 enteric viral serotypes that are acknowledged to infect humans, and the major illness associated with HEV is gastrointestinal illness [50]. HEV have also been implicated in acute illnesses, such as meningitis, conjunctivitis, hepatitis, poliomyelitis, respiratory diseases and severe fever [50,51]. These groups of viruses are easily transported and transmitted via adsorption phenomena, in the following way: from one contaminated water point to another (especially through the fecal–oral route) [50,52,56], from wastewater treatment plants’ effluents [51,57,58], due to agriculture runoff [51,55], leaking septic tank systems [51,59], and recreational and food products [51,60]. Although HEV cannot reproduce themselves outside their host’s cells, they still have the potential to stay alive for extended periods of time within the aquatic environment [50,61]. Moreover, some serotypes have a strong resistance to chlorine disinfection, which is the most common treatment used at many wastewater treatment facilities [50,53]. The resistance towards chlorine treatment may be due to their high resistant protein coat. However, after treatment, the effluents are released into the aquatic ecosystems, as they are the main sources for drinking water, aquaculture and recreation [61]. The outbreak of HEV disease in both developed and undeveloped nations, has been globally documented by the World Health Organization (WHO) [62]. In the United Kingdom for instance, the effects of these outbreaks has led to a huge strain on the healthcare system, economic burden, and also decreased productivity in affected persons [63]. Table 1 shows some known and identified HEV that are a threat to the global aquatic ecosystem.

**Figure 2:**
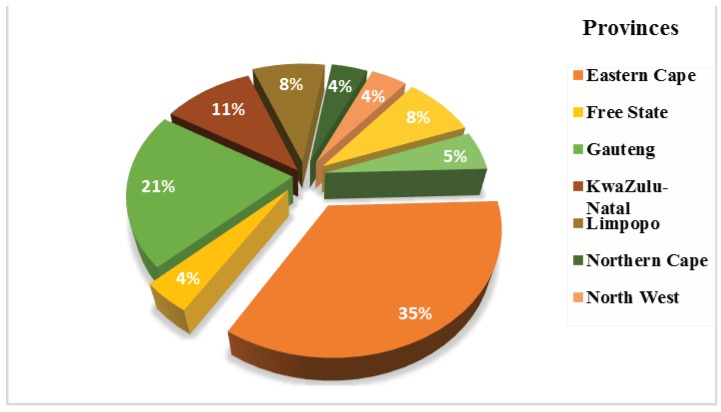
Chart showing the percentage of HEV study in different province of South African aquatic ecosystems between 1993–2015 [58,69,73,74,77].

In South Africa, hepatitis A, adenoviruses, astroviruses, noroviruses, enteroviruses, rotaviruses and bacteriophages, have been detected in surface water [50,68,69], wastewater treatment plants [70,71], and in treated drinking water sources [59,70,72,73] in some provinces in South Africa. The identification and quantification of HEV in South Africa was mostly done using conventional and traditional methods in both clinical and environmental samples. Figure 2 shows the different provinces in South Africa where HEV have been studied and identified in different aquatic environments. Over the years, Taylor and his co-workers have extensively investigated the consecutive outbreaks and presence of some HEV outbreaks from some patients through the exposure to surface waters, dams, WWTPs [74,75,76,77]. Techniques such as metagenomics, is still an emerging technique for the identification and diversification of HEV in both environmental and clinical samples in South Africa. There is little knowledge pertaining to the viral content and diversity in wastewater systems in South Africa, which demonstrates the need to survey viral communities using metagenomics. Based on the limitations of the existing molecular methods that target specific viruses, and specific bacterial indicators, new methodologies such as metagenomics are vital for the identification of unique or unlooked-for viruses in the aquatic ecosystems.

## 2. Conventional Methods for the Identification of HEV in Environmental Samples

Sample volume in addition to sampling method are the most challenging steps required in the identification of HEV in environmental samples [78,79]. For the initial concentration of viruses, the adsorption elution principle has been widely applied for the primary concentration of enteric viruses from water, based on the fact that viruses mechanisms are linked to the surface charge [80,81]. In line with the distinguishing viral particle surface capabilities, they have the potential to eagerly adsorb to a number of materials [82]. However, in recent years, a wide range of concentration procedures and techniques have been implemented for the primary and secondary concentration of viruses in water samples. This entails the adsorption of virus-related particles or phages onto the surface of a filter membrane, through the interaction of electrostatic charges, followed by elution with the appropriate buffer system [82,83,84,85,86,87]. Alternatively, the concentration of viral particles could also be based upon size exclusion of the particles, rather than the electrostatic interactions of the filters on the viruses [82], with varying adsorbent material and elution buffers. In Africa, some of these concentration techniques have been used and reported [59,70,72,88]. Table 2 provides a short summary of the conventional and improved concentration procedures used for the recovery of HEV in environmental samples.

### 2.1. Culture Based Methods

In vitro growth methods such as cell culture are the most pronounced traditional standards used to identify and detect the occurrence of HEV in environmental samples [82,98,99]. Cell culture is a technique whereby a microorganism’s cells are grown at a carefully controlled condition outside of the living animal [100]. It is a very time consuming, laborious and expensive approach that usually demands prior knowledge of the targeted species [51,70]. The limiting factor with this method is that there are some viral species that are not capable of producing any cytopathic effect when propagated on a cell line [51]. HEV detection has also been explored using the integrated cell culture polymerase chain reaction (ICC-PCR), this technique has also been used for the discovery of HEV in ecological samples [65,101,102]. The merit of this technique is that it gives room for several modifications of the protocols, enhanced the direct analysis and monitoring of HEV in environmental samples [103,104,105,106].

**Figure 3:**
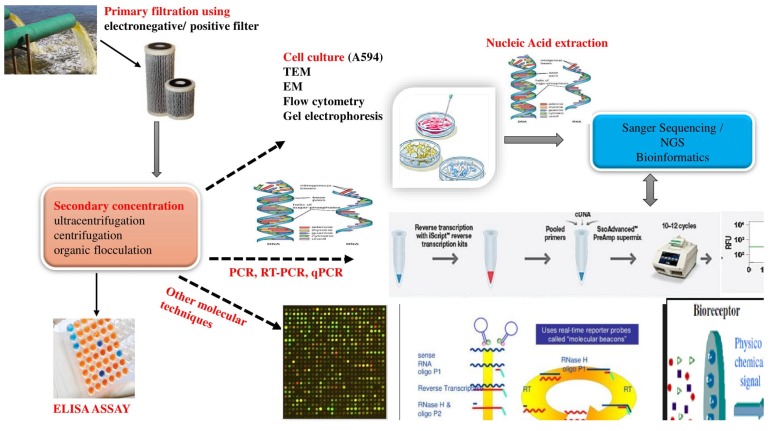
Schematic illustration of various molecular techniques applied for the identification of HEV from different environmental samples.

Epifluoroescence and transmission microscopy, is another type of conventional technique that has been explored for the abundance, morphological and enumeration studies of viral entities within the aquatic environments [107,108]. Here, the virus-like particles are counted using fluorescent nucleic acid stains through visualisation [107,108,109,110]. Flow cytometry and vortex flow filtration (VFF) have also been used for the quantification and counting of virus-like particles and prokaryotes in aquatic environments [98,111,112]. Figure 3 exhibits the numerous molecular approaches that have been used in the diagnostics and identification of HEV in environmental samples.

### 2.2. Polymerase Chain Reaction Methods (PCR Assays)

Polymerase chain reaction (PCR) is a sensitive conventional assay technique that is used on targeted amplification of the viral DNA or RNA over a range of magnitude to produce thousands or millions of copies [51,106]. PCR methods are designed to amplify a single specific nucleic acid sequence a million times under three distinctive steps that include denaturation, annealing and extension. For denaturation to take place, the target DNA is subjected to a high temperature in other for the DNA strands to be separated. Annealing of the primers to the target DNA allows the DNA to polymerase and selectively amplify the target DNA at a lower temperature [51]. PCR assays are very sensitive, highly specific, and particularly attractive for detection of non-cultivable infectious agents thereby making it an attractive method for the detection of target pathogens [51]. A comprehensive array of PCR systems exists for rapid detection and confirmation of the presence of HEV in different environmental samples. These samples include water sediments [113,114], wastewater treatment plants (WWTP) [59,115], treated and untreated sewage [115,116], groundwater [117], and surface water [69,102]. A wide range of primers have been designed for the precise detection of many HEV and an immediate overview of these is presented in Table 3. The chief limitation of the PCR techniques is that they are incapable of distinguishing between active and inactive targets, and are found to be prone to inhibition due to the interaction with DNA or interference with the DNA polymerase which increases false negative results. In addition, different primer sequences make it inappropriate for use, especially with the discovery of unique viruses. Previous information of the viral sequence is, therefore, a pre-requisite for any PCR reaction. Various modifications of the PCR assay have been used for detection of HEV, and they include the nested [118], multiplex [119,120,121], real time [106], and reverse-transcription polymerase chain reaction [118], all displaying their own merits and demerits.

The presence of norovirus, astrovirus, enterovirus have been established have been established in surface water, ground water and wastewater samples via multiplex and nested PCR [51,120,136]. Other modified PCR techniques developed are the reverse-transcriptase polymerase chain reaction (RT-PCR) and real-time or *quantitative polymerase chain reaction* (*qRT-PCR*). The RT-PCR are able to amplify and detect HEV viruses that possess only the RNA genomic information [27,49,69,89,109,110,111,112]. These techniques has been implemented for the identification of different groups of the HEV in various environments [78,83,84,106,117,137,138,139,140,141,142]. These techniques also offer better rates of detection, and great sensitivity and accuracy. In addition, they are precise, they reduce experiment time and the possible source of contamination is reduced [51,78]. A summary of the numerous molecular techniques, principles, merits and limitations is presented in Table 4. 

### 2.3. Viral Metagenomics

**Figure 4:**
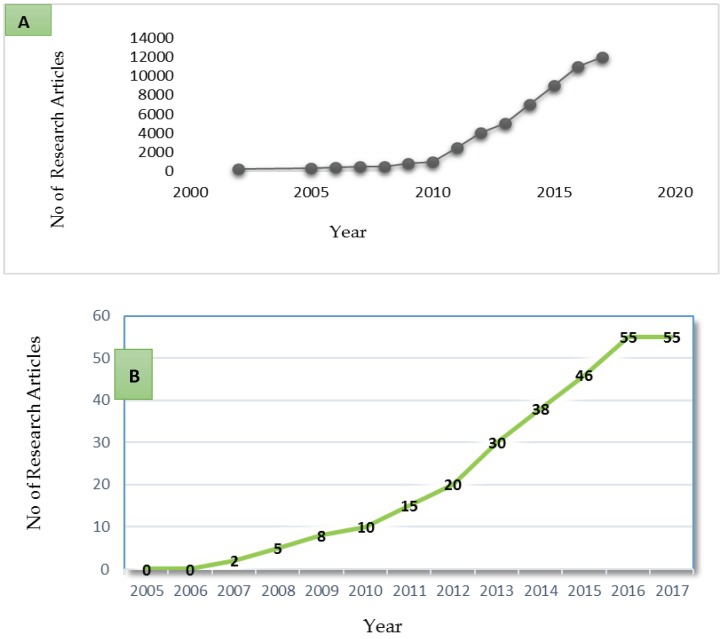
(**A**) Overview of the research publications of viral metagenomic studies around the world, (**B**) Overview of the publication of viral metagenomic studies in Africa.

Viral metagenomics is a modern genomic technique used for studying viral communities in their natural habitat, without the isolation and laboratory cultivation of single species [170,171,172,173]. The sequencing of the genomic DNA information using metagenomics can be achieved either through the PCR amplicon sequencing or via shotgun metagenomics. The PCR amplicon approach, is mainly used for targeted species, the identification and characterization of the specific genomic regions is done through the use of specific primers [174,175]. The second approach, shotgun metagenomics, is a technique whereby unculturable and difficult microbes are analysed and studied extensively without prior knowledge of the state of these communities [174,176]. There has not been an individual gene marker that is peculiar to most viral genomes, like the 16S RNA used to denote the bacteria genome [1,171], hence, this has limited the understanding and investigation of viruses by amplicon sequencing and ribosomal DNA profiling [1]. Studies on viral metagenomic have revealed that a lot of the generated sequences are not similar or matching to known viruses, hence the need for viral metagenomic analysis in the virology field [171,177]. Specifically, viral metagenomics has provided the detection of viral species presumed to be a potential threat to human health [130,178], means for virus discovery [179], and the characterization of the viral population [171,180]. Figure 4A, B provide an overview of the number of research articles on metagenomic studies on human virome in diverse parts of the world. They also indicate how the number of research articles has risen from around 200 articles in 2002, to more than 12,000 articles in 2017. Due to this, more metagenomic datasets of viruses have been established [171,177]. Africa is still far behind in terms of research articles being produced, with approximately 50 articles available, to date.

The first-generation sequencing is a chain-termination technique, where sequencing is achieved by the selective incorporation of chemical analogues of deoxyribonucleotide triphosphates (dNTPs), the monomers for DNA strand synthesis [181,182], with an approximate reads of approximately 1200 bp long [183]. This technique has been used to characterize the presence of the different groups of human adenoviruses (HAdVs) in environmental samples [184]. The main setback of this technology is that it is a low throughput, thereby limiting it as a means for diagnosis, and is labour intensive and slow [181,183]. In 2004, the revolution and activation of an improved sequencing knowledge began through the introduction of the second-generation sequencing platform [181,185]. The second-generation platform includes 454 Roche platform, Ion Torrent Personal Genome Machine, AB SOLiD and Illumina Solexa sequencers [22,23,181,185,186]. The 454 sequencing platform has been used to examine the diversity of human RNA viruses present in Lake Needwood, a freshwater lake in Maryland, USA, with results indicating the presence of four different types of viruses [187]. Likewise 454 platform was able to detect and study the dominant DNA and RNA viral species in reclaimed water, the study showed that both the reclaimed and portable water was dominated by phages [188], it has also be used as a monitoring tool for identification of viral agents of animal, plant and human diseases in freshwater samples [189]. Ion Torrent platform has also been explored for the sequencing and microbial profiling of multiple viral groups from animal samples and sediments from the Athabasca River [190,191]. The Illumina Solexa technology system seems to be the most favoured platform over other existing second-generation platforms. The sequencing of microbes is based on the sequence by synthesis (SBS), with upgraded system versions [22,185,192]. Illumina systems have been used to sequence viruses from both clinical and environmental samples [193,194]. Table 5 shows the strength and weakness of the second- and third-generation platforms. The rudimentary workflow for second-generation sequencing is shown in Figure 4.

Recently, the emerging third-generation sequencing technologies that are being introduced in the genomic scientific world are the Pacific Biosciences Single Molecule Real Time (SMRT) sequencing, Nanopore sequencing by Oxford Nanopore, and the Helicos TM Genetic Analysis System [23,169,186,195,196]. The technology has the potential of generating high read lengths of up to 100,000 bp within hours, and is very expensive to acquire [186,195,196]. The most recent third-generation technology is Nanopore Technology, which involves the use of a small device or membrane with a pore size of approximately 1.5–2 nm [186]. The distinguishing feature of all the third-generation sequencing platforms is that the technique does not require an amplification step during the library preparation [196]. In addition, the read lengths are between 25–15,000 bp, with a run time of approximately 30 min, when compared with the second-generation platforms [195,196]. Pacific Biosciences Single Molecule Real Time technologies has explored some microbial populations [197]. Currently, these technologies are being developed and upgraded, but they have not been exclusively explored to the fullest for the determination and analysis of the HEV, probably due to cost of set-up and lack of technical skills.

## 3. Metagenomics and Its Application in Africa

In certain countries, viral metagenomic studies have increased gradually [171,201,202]. It is emerging as an alternative technique for viral identification, diversity and abundance, in a range of environmental samples which includes the ocean environment [48,170,203], surface freshwater bodies and lakes [187,204], ballast water [202], wastewater plants [205,206], reclaimed water [188], the atmosphere [207], plants [208], aquaculture [209], and in clinical samples such as feces [210], blood [211], and in some animals [212]. In the face of the advances in the biological world, where the cost of sequencing is gradually reducing, developing countries such as South Africa are still a long way from benefiting from the technology. Over the years, environmental metagenomic studies in South Africa have focused mainly on studying diversity and abundance of bacteria in different aquatic ecosystems and extreme environments [213].

In 2015, Tekere and co-workers carried out a metagenomic analysis study in a thermal hot spring in Limpopo. The aim was to define the genetic and phylogenetic diversity of thermophiles in this environment. The community composition, distribution and abundance of the thermophiles living in the different hot spring waters, and biofilms of South Africa, were assessed [149,213,214,215]. In addition, the abundance of halophilic bacteria were also identified from a salt pan in the Limpopo province [216]. In 2018, Abia and co-workers used metagenomics to analyse the functional profiles of some bacterial populations in sediments as well as in surface water samples. It was observed that the abundance and diversity of bacterial is attributed mainly to the occurrence of an unapproved informal settlement with poor infrastructure. The functional profiling revealed that bacteria could be a possible pathway in human diseases [217]. In addition to the natural environments, man-made extreme environments such as industrial wastewater, was also explored for bacteria diversity [218].

Metagenomics is progressing slightly in Kenya, since it has been observed that arthropods—which are referred to as blood-feeding agents for viruses—could cause an exceptional health concern [219]. The intercontinental virome diversity studies on the culex mosquitoes were done using samples from Kenya and China and analysed using NGS. The study revealed that mosquitoes are vital vectors as well as the fact that viruses are harbored by these arthropods [219]. The study also indicated the presence of some specific vertebrates, invertebrates, plants, and protozoa as well as uncategorized assembly of viruses [219]. Another part of Africa that metagenomics is also gaining momentum in is Namibia. Metagenomics has been employed to better understand virus abundance, ecology and diversity in the soil samples [220]. The enumeration of these viral particles on different types of soils has shown that viral abundance can range from 1.5 × 10^8^ to 6.4 × 10^8^ per gram of soil [220,221]. NGS has also been used to determine the diverse ecological patterns in the Namib Desert, the cold Miers Valley, and the Antarctica hyper arid deserts, so as to understand the response to, and microbial adaptation to, environmental stressors [222]. Likewise, comparative metagenomic studies have been conducted on the mechanisms that are likely responsible for the stress response in hypoliths in extremely hot hyper-arid desert soils [223]. In Kampala, Uganda, the diversity and richness of some HEV was investigated from wastewater samples and surface water using viral metagenomics. In this study, numerous human and vertebrate viruses were discovered, such as Herpesvirales, Iridoviridae, Poxviridae, Circoviridae, Parvoviridae, Bunyaviridae from the effluent samples [178]. Through the study, it was also established that the discharge from the wastewater treatment plant appears to influence the quality of the surface water through high viral concentrations levels. Although in this study, only the sampling and filtering of the water samples was done in Uganda, the NGS analysis, and data interpretation of the sample was done at Michigan State University in the United States. This was probably due to the fact that most of the infrastructure, cost and manpower associated with the metagenomic study and pipeline were not available.

In South Africa, a study of viral diversity using metagenomics has not been explored to the fullest, except in few environments. In Kogelberg Biosphere Reserve in South Africa, the unique plant viral biodiversity was explored in a vegetation in the western province using metaviromic technique. The recovered DNA from the soil samples was sequenced under the Illumina Platform with some bioinformatics analysis carried out which detected biodiversity among the Caudovirales group [224].

The functional and phylogenetic analysis of the metaviromes revealed a high percentage of phages while distinct viromes from known isolates were left. New and emerging phage related protein sequences were also identified in this research study, thereby presenting a prospect for more research studies in such environments to explore more viral diversity using metagenomics. 

Metagenomics was also explored in South Africa, in Western Cape province, to determine the unique interaction of viruses’ diversity in an African hot spring community; this was achieved via electron microscopy and sequencing [225]. In this study, the metaviromes analysis was able to detect the presence of salterproviruses using a polymerase B gene phylogeny [225]. The diversified presence of phages, as well as novel archaea viruses, was also discovered in the hot spring. Likewise, a research group in the Eastern Cape province employed the approach of viral metagenomics to screen, identify, and recover, the prevalent species of Human Adenovirus (HAdV) present in sewage and mussel samples, which are associated with human infections [226]. In this study, the metaviromes indicated the predominant presence of HAdV-17 in mussel samples. This is an indication that it is not only the environmental samples that should be the most important priority; both food products and clinical samples should be screened thoroughly. The manifestation of HAdV-D17 in the seafood samples raises an alarm round the ecological health state of the river as well as the extent of contamination existing in the Swartkops River estuary [226]. Table 6 demonstrates the trends of the metagenomics approach using different sequencing platforms in Africa.

## 4. Open Research Work and Implications for Environmental Genomes

More insight into virology ecology has expanded since the commencement of viral metagenomics. At present, in South Africa, conventional molecular techniques have mainly been used in the isolation, quantification and identification of HEV. In all these conventional approaches used thus far, our knowledge of the different species of viruses in the environment has been limited. More information about the occurrence, abundance, diversity and ecological richness of these microbes remain unexplored due to lack of skills and technology. Characterization of viral communities through conventional methods or protocols is often biased, as they do not allow for total viral community analyses. Some of these techniques are peculiar to a gene or organism, tedious and specific since no specific molecular assay has the potential to determine all viruses present in a sample in one single run. NGS has received huge success and application in viral ecology in various matrices, where other techniques have had setbacks. Based on literature and scientific reports, identification of HEV using metagenomics is still an upcoming approach in resource-poor settings like underdeveloped or developing regions. The non-stop monitoring of bio-indicators in wastewater systems using metagenomics could also attribute to evaluating the distribution patterns of viral infections, as well as the microbial risk assessment, which can make available early advice of any potential disease outbreaks. The South African aquatic systems have the prospect of an almost unimaginable microbial diversity, despite the water scarcity syndrome been experienced in recent years. Techniques such as viral metagenomics can be used to improve surveillance of viral pathogens, to understand the evolution and diminishing viral species due to climate changes, and for diversity in food security and public health.

## 5. Conclusions and Future Perspectives

Since the introduction of metagenomics and NGS, the field has gained momentum, giving room and opportunity for the characterization of all possible microbes in a sample. Since there is not much development in the areas of cutting-edge technologies in developing nations, the quest for information regarding the state of our water systems continues to deteriorate.

Emerging and recurring viral species may not be the only setbacks facing developing countries, but a problem that the entire world faces. This is due to the fact that these viruses have a mysterious way of contaminating and polluting the world’s entire aquatic ecosystem. It is proposed that the investigation about the prevalence of possible microorganisms within the aquatic system is essential because diverse activities are carried out in various parts of the world. The relatively high cost of modern molecular technologies, as well as computational human expertise for the analysis of the data generated, have greatly contributed to the slow growth of the viral microbial ecological research community in Africa. NGS is undeniably a key technology; however, the implementation of this technique is still a challenge in Africa. A wide range of challenges are defying researchers in Africa, such as limited scientific resources, limited human skills, insufficient training and lack of access to genome sequencing facilities. In addition, we recommend that more energy should be directed towards instituting more water and safety programmes in emerging nations, as this may help to break the barriers and restrictions that are swallowing up the scientific community.

## Figures and Tables

**Table 1:** Human enteric viruses (HEV) that have been identified in various aquatic environments.

Family	Genus	Collective Names	Adverse Effects	References
*Picornaviridae*	*Enterovirus (ssRNA)* *Hepatovirus, Hepevirus,* *Sapovirus (ssRNA)*	Poliovirus, Echovirus, Coxsackievirus A, B Hepatitis A, E Sapporo-like virus	Meningitis, Paralysis, Myocarditis, respiratory infections, gastroenteritis Infectious Hepatitis	[51,52,62,64,65,66,67]
*Reoviridae*	*Rotaviridae (dsRNA)*	Human rotavirus	Gastroenteritis	[51,52,62,64,65,66,67]
*Adenoviridae*	*Mastadenovirus (dsDNA)*	Human Adenovirus	Conjunctivitis Gastroenteritis Respiratory diseases	[51,52,62,64,65,66,67]
*Caliciviridae*	*Calicivirus (ssRNA)* *Polyomavirus (dsDNA)*	Human calicivirus Norwalk virus Polyomavirus	Gastroenteritis, Fever Progressive Multifocal leukoencephalopathy, Urinary tract diseases	[51,52,62,64,65,66,67]
*Astroviridae*	*Mamastrovirus* *Parovirus*	Human astrovirus Human parvovirus	Gastroenteritis	[51,52,62,64,65,66,67]
*Coronoviridae*	*Coronavirus (ssRNA)*	Human coronavirus	Gastroenteritis Respiratory diseases	[51,52,62,64,65,66,67]
*Circovirus*	*Torovirus (ssDNA)*	Human Torovirus	Gastroenteritis	[51,52,62,64,65,66,67]

**Table 2:** Different concentration techniques used for the concentration, recovery and isolation of viruses in environmental samples.

Virus	Technique	Advantages	Disadvantages	References
*Enterovirus*	Membrane adsorption technique	Simple, speed, sensitive	Low efficiency of virus adsorption Easy clogging of membrane filter	[89]
*Enterovirus*	Aqueous polymer two phase separation	Simple and cost effective Requires small sample volumes	Limited serotypes identified Inhibitory action of salts	[82,89,90]
*Poliovirus, Herpesvirus, Echovirus*	Adsorption to precipitable salts, iron oxide, and polyelectrolytes	Requires large sample volume, simple, time effective	Specific to certain viruses and water samples	[81,82]
*Poliovirus*	Soluble alginate filter	Simple, Non-cytotoxic	Clogging of filters, Pre-filtration required, time consuming	[81,82,89]
*Poliovirus*	Continuous-flow ultracentrifugation	Opportunity for diversity	Expensive instrumentation, time consuming	[81,82,89]
*Bacteriophage*	Forced-flow electrophoresis and electro-osmosis	Small sample volumes, less processing time	Small sample volume	[82,89]
*Enterovirus*	Hydro extraction	Good recoveries	Small sample volume	[82,89]
*Poliovirus*	Gauze sampler	Large sample volume, cost effective	Low efficiency Minimal recovery of viruses	[82,89]
*Poliovirus, Norovirus, Enterovirus*	Electropositive Filtration	Large sample volume, pre-conditioning step not required, Cost effective	Not effective for selected environmental samples including marine water and sediments, expensive	[82,83,91,92]
*Poliovirus, Echovirus, Reovirus, Coxsackievirus*	Electronegative Filtration	Varieties of adsorbent materials, available, High recoveries	Conditioning of large volumes of water is difficult, Acidification protocol may lead to the formation of precipitates, Filter clogs easily, Expensive	[80,82]
*Poliovirus, Enterovirus, Rotavirus*	Glass wool	Less expensive, Pre-conditioning of water sample is not required	Not suitable for large sample volume	[70,71,93,94]
*Poliovirus, Echovirus, Hepatitis A*	Ultrafiltration (Tangential flow, Dead-end flow, Vortex)	No pre-conditioning steps required	Expensive, retreatment of fibres important	[82,95,96]
*Calcivirus, Hepatitis A*	Ultracentrifugation	Less time consuming, Large volumes of water are concentrated to millilitres	Clarification step required, Loss of viruses through the use of membrane filters, expensive	[82,89,97]

**Table 3:** Review and summary of published primers for PCR Assays.

HEV	Primers and Labelled TaqMan Probes	Target Region	References
Hepatitis A virus	HAV68 (F): 5′-TCA CCG CCG TTT GCC TAG-3′ HAV240 (R): 5′-GGA GAG CCC TGG AAG AAA G3′ HAV150 (P): 5′-FAM-CCT GAA CCT GCA GGA ATT AA-MGBNFQ-3′	capsid gene VP1/P2B	[69,73,116,122,123]
Enterovirus	EV1 (F): 5′-CCCTGAATGCGGCTAAT-3′ EV1 (R): 5′-TGTCACCATA AGCAGCCA-3′ EV-BHQ (P): 5′-FAM- ACGGACACCCAAAGTAGTCGGTTC-MGBNFQ-3	5′ Non-coding region	[57,58,69,73,124,125]
Rotavirus	JVK (F): 5′-CAGTGGTTGATGCTCAAGATGGA-3′ JVK (R): 5′-TCATTGTAATCATATTGAATACCCA-3′ JVK (P): 5′-FAM-ACAACTGCAGCTTCAAAAGAAGWGT-MGBNFQ-3′	NSP3 gene	[69,73,126]
Noroviruses GI GII	JV13I (F) 5′-TCA TCA TCA CCA TAG AAI GAG-3′ JV12Y (R) 5′-ATA CCA CTA TGA TGC AGA YTA-3′ JV13I (F) 5′-TCA TCA TCA CCA TAG AAI GAG-3′ G1 (R) 5′-TCN GAA ATG GAT GTT GG-3′ JV12Y (F) 5′-ATA CCA CTA TGA TGC AGA YTA-3′ Noro11(R) 5′-AGC CAG TGG GCG ATG GAA TTC-3′	Polymerase region	[73,127]
Adenoviruses	JTVX(F) 5′-GGACGCCTCGGAGTACCTGAG-3′ JTVX(R) 5′-ACIGTGGGGTTTCTGAACTTGTT-3′ JTVX(P):5′-FAM-CTGGTGCAGTTCGCCCGTGCCA-MGBFQ-3′	Hexon gene	[58,128,129]
Astrovirus	HAst.(F): TCAACGTGTCCGTAAMATTGTCA HAstV. (R):TGCWGGTTTTGGTCCTGTGA HAstV.probe1(FAM): CAACTCAGGAAACAGG HAstV.probe2 (FAM): CAACTCAGGAAACAAG	ORF 1b-VPg region ssRNA	[130]
Sapovirus GI, II and IV	Sapo (F) A: ACCAGGCTCTCGCCACCTA Sapo (F) B: ATTTGGCCCTCGCCACCTA Sapo (R): GCCCTCCATYTCAAACACTAWTTT Sapo.probeA (FAM) CTGTACCACCTATGAACCA Sapo.probeB (FAM) TTGTACCACCTATGAACCA Sapo.probe C (FAM) TGTACCACCTATAAACCA Sapo.probe D (FAM) TGCACCACCTATGAAC	RdRp-VP1 region	[130,131]
Salivirus	F: 5′-TCTGCTTGGTGCCAACCTC-3′ R: 5′-CCARGCACACACATGAGRGGATAC-3′ Probe: 5′-FAM- TGCGGGAGTGCTCTMGB- NFQ-3′	VP1 region or 3CD region	[132,133]
Klassevirus	KLA-F; 5′-TCTGCT TGGTGCCAACCTC-3′ KLA-R; 5′-CCARGC ACACACATGAGRGGATAC-3′ KLA-TP; 5′FAM-TGCGGGAGTGCTCT-MGB-NFQ-3′	VP0/VP3 regions	[133]
Human Parechovirus	F: 5′-CCA AAA TTC RTG GGG TTC-3′ R: 5′-AAA CCY CTR TCT AAA TAW GC-3′	VP1 capsid gene or 3CD region	[134,135]
Aichi virus	F: ACA CTC CCA CCT CCAGCC AGT A R: GGA AGA GCT GGG TGT CAA GA	3CD junction region	[134,135]

**Table 4:** Summary of the Pros and Cons of molecular methods for HEV identification.

Technique	Principle	Advantage	Disadvantages	References
Cell culture	Cytopathic effects potential for viruses	Direct isolation of a variety of cultivable viruses to high titres	Highly skilled Requires controlled conditions Expensive and time consuming	[51,99,105]
Electron microscope transmission electron microscopy	Electron beam used to illuminate viruses. Counting of the viral particles and morphology	Prior knowledge of organism not required DNA provides high resolution image	It requires technical skills and expertise Poor detection limit High concentrations High cost of maintenance and training of the instrument	[97,109,110,143]
Flow cytometry	Direct and rapid assays for the determination of cell numbers and morphology	High speed and velocity	Skill generation and refrigeration a pre-requisite, expensive	[112,144]
Vortex flow filtration	Counting and quantifying virus-like particles	High recovery Reduces filter clogging	Expensive method	[107,112,144]
PCR Assay	Amplification assays based on specific primers and enzyme to generate more copies of DNA	Sequence dependent Cost effective High sensitivity and specificity	Cannot detect new viral species Risk of contamination False positive results	[51,66,106]
ICC-PCR	Viral particle is amplified via host cell assays	Less vulnerable to PCR inhibition Identify non-cytopathic viruses	Does not detect non-culturable viruses, Requires multiple cell lines Time consuming, More costly than direct PCR detection	[51,66,105,106]
Multiple PCR	Simultaneous amplification of sequences of several pathogenic microorganisms in a reaction mixture	Sequence dependent, Cost and time effective, High sensitivity and specific	Cannot detect new viral species Challenges with optimisation and sensitivity for all targeted species Contamination Non-specific amplification in environmental samples	[51,106,136,145]
Nested/Semi Nested PCR	Distinct pair of primers amplifies enormous region of DNA The amplified PCR product is now used as a template for the next round of amplification	Increased sensitivity	Potential risk of contamination and carry-over	[51,106,120,146]
RT-PCR (Reverse-transcriptase PCR)	Amplification is achieved by converting DNA to complementary DNA (cDNA) in a reverse transcription procedure	Speed sensitivity Contamination Specificity Repeatability	Sequence knowledge is a perquisite, expensive, Possible reaction inhibition, and there is a need for experts for the interpretation as well as the accuracy of results	[51,66,78,106,137,147,148,149]
qRT-PCR (quantitative real-time PCR)	Quantifies and measures amplification of DNA using dyes or fluorescent dyes or probes	Elimination of gel electrophoresis applicable for both culturable and unculturable microorganisms		
Microarray technology	Detection is done by means of radio-labelled probes or fluorescent tags	Known viral sequences	Expensive Reproducibility test results are poor	[150,151,152,153,154]
NASBA	Isothermal amplification of RNA	Sensitive, rapid simple Resistant to matrix influence	Can be used only for organisms, which are already known	[106,155,156,157,158,159,160,161]
Immunology-based method	Formation of antigen—antibody through recognition and binding	High sensitivity Specificity speed Easy automation and equipment	QC assurance dependent Risk of interferences Expensive	[162,163]
Biosensor-based methods	analytical device that identifies analytes via an electrical signal	Detects non-polar molecules High specificity Reaction time is short	Relies on specific antibodies or DNA Probes Necessary chemical inactivation for the recognition sites	[164,165,166,167,168]
NGS	Parallel sequencing of multiple small fragments of DNA to determine its sequence using high-throughput instrumentation	Fast and easy to approach for DNA sequencing Large sequencing data per run	Expensive equipment	[169]

**Table 5:** Summary of the various features of the different second-generation platforms indicating strength and weakness.

Platform	Amplification Technique	Chemistry	Read Length	Output and Duration	Advantages	Disadvantages	References
Roche 454	Emulsion PCR	Pyro-sequencing	400–700 bp	100–700 Mb 10–23 h	Long read length, short run times	High error rate	[22,23,186,196,198,199,200]
AB SOLiD	Emulsion PCR	Ligation	35 bp	80–360 Gb between 6–8 days	Low error rate	Short reads Long run time	[22,23,185,186,195,196,199]
Ion Torrent (PGM)	Emulsion PCR	Proton detection	100–400	100–64 Gb for 2–7 h	Less sequencing time, reduces costs	Short reads Homopolymer errors	[22,186,192]
Illumina Solexa (MiSeq, HiSeq)	Bridge PCR	Reversible terminators	100–300	600 Gb 5 h to 3 days run	High throughput, Cost and time effective, minimal error rate	Short reads Decrease in quality of reads towards the ends	[22,186,192]
Pacific Bioscience (SMRT)	Single molecule real time (SMRT)	Fluorescently labelled nucleotides	4000–5000 nts	200 Mb–1 Gb generated under few hours	Data generation is monitored in real-time, Accurate	Expensive, high error rates	[22,23,186,195,196]
Helicos TM Genetic Analysis System	non-amplified DNA templates	Fluorescently labelled nucleotides	24–70 bp	35 Gb for a few hours	Accurate	Expensive, low data output	[23,186,196]
Oxford Nanopore (MinION)	Single molecule real time (SMRT)	Reversible terminators	90 Mbp of data with 16,000 reads	6 kb–60 kb	Accurate	Expensive, high error rate, low throughput	[22,23,186,195,196]

**Table 6:** Recent studies and application of metagenomics in some African countries.

Country	Microbe	NGS Platform	Environment	References
South Africa	Bacteria thermophiles	Roche 454	Hot spring	[149]
South Africa	Bacteria	Illumina MiSeq	Surface water Sediments, Industrial wastewater	[215,217]
Namibia	Virus		Soil, deserts	[220,221]
Kenya	Mosquito	Illumina	Clinical sample	[219]
South Africa	Virus	Illumina	Hot spring	[225]
Uganda	Viruses (HEV)	Illumina	Surface water, WWTP	[178]
South Africa	Viruses (HADV)	Illumina	Sewage Mussels	[226]
South Africa	Viruses (Caudovirales, phages)	Illumina MiSeq	Soil	[224]
